# Perspectives of urban Ghanaian women on vasectomy

**DOI:** 10.1186/s12978-017-0286-5

**Published:** 2017-02-08

**Authors:** Ofeibea Asare, Easmon Otupiri, Joana Apenkwa, Rose Odotei-Adjei

**Affiliations:** 1 School of Public Health, Kwame Nkrumah University of Science and Technology, Kumasi, Ghana; 2 Catholic University College of Ghana, Fiapre, Ghana

**Keywords:** Urban Ghanaian Women, Male partners, Vasectomy, Perspectives, Uptake

## Abstract

**Background:**

Advocacy for male involvement in family planning has been championed over the years after the 1994 International Conference on Population and Development (ICPD). There are a few contraceptive methods for men, and vasectomy uptake has been identified as one of the indicators of male involvement in family planning. Vasectomy also known as male sterilization is a permanent form of contraception. It is a generally safe, quick, easy, effective surgical operation with rare complications to prevent release of sperm. The study explored the vasectomy perspectives of urban Ghanaian women.

**Methods:**

A qualitative approach was used and five focus group discussions were held with women in urban Accra. The study was conducted in the five sub-metropolitan areas of the Accra Metropolitan Health Directorate from September–October 2013. Participants were adult and young adult women who are members of organized groups and unions. Data were analyzed manually after transcribing and coding and themes were sorted using thematic version 0.9.

**Results:**

Both adult and young adult participants regarded vasectomy as an easy way for male partners to become promiscuous and cheat on them (women) because the operation renders males incapable of having a child; promiscuity could lead to the women contracting sexually transmitted infections including HIV/AIDS. They were also skeptical about vasectomy and the possibility that it could damage the sexual organs of their partners and affect their sexual relationships. The uptake of vasectomy will not benefit a new wife in case of divorce or death of a previous wife. Some women would allow their partners to undergo the procedure only if both of them will benefit health-wise and also if it would reduce the financial burden on the family.

**Conclusion:**

The women held mixed perceptions; both negative and positive views were shared on vasectomy uptake. The views were predominantly negative, and they regarded vasectomy as an unacceptable method of contraception. The women virtually had no reasons to encourage their partners to undergo a vasectomy. In order to increase vasectomy uptake in Ghana, innovative efforts to address the misconceptions and superstitions surrounding vasectomy should take centre stage; appropriate and targeted messaging during integrated health services delivery and social/health campaigns would be a good starting point.

**Electronic supplementary material:**

The online version of this article (doi:10.1186/s12978-017-0286-5) contains supplementary material, which is available to authorized users.

## Plain English summary

There are a few contraceptive methods for men, and vasectomy uptake has been identified as one of the indicators of male involvement in family planning. Vasectomy also known as male sterilization is a permanent form of contraception. It is a generally safe, quick, easy, effective surgical operation with rare complications to prevent release of sperm. The study explored the vasectomy perspectives of urban Ghanaian women.

Five focus group discussions were conducted among young adults and adult women who are members of organized groups in five sub-metropolitan areas in Accra. In total forty-eight (48) participants partook in the study; twenty-three (23) young adult women and twenty-five (25) adult women.

Both adult and young adult participants regarded vasectomy as an easy way for male partners to become promiscuous and cheat on them which could lead to the women contracting sexually transmitted infections including HIV/AIDS. They were also skeptical about vasectomy and the possibility that it could damage the sexual organs of their partners which will affect their sexual relationships. Some women would allow their partners to undergo the procedure only if both of them will benefit health-wise and also if it would reduce the financial burden on the family. From the responses three themes were identified from the study; social perspectives, health perspectives and economic reasons.

The perceptions held by women on vasectomy uptake were predominantly negative. If vasectomy uptake is to increase in Ghana, there still remains a huge hurdle to overcome. Vasectomy advocates and family planning programmers could place emphasis on the economic gains it brings to individuals, households and nations.

### Background

“Vasectomy is not an end in itself but a beginning of taking responsibility for the family’s welfare” [1]. The 1994 International Conference on Population and Development (ICPD) held in Egypt identified male involvement in contraception as vital to women’s health [2]. Advocacy for male involvement in the use of family planning methods has been promoted over the years [3]. This is to emphasise the role of men in helping make the ‘contraceptive burden’ lighter for women [4]. From the ICPD report, it was obvious that an increase in male sterilization is a key indicator for measuring male involvement in family planning. Vasectomy, male condoms, withdrawal and abstinence are the common contraceptives available to men. Vasectomy is a permanent form of contraception for men. It is the safest, simplest, least expensive and yet equally effective form of modern contraceptives. It is a simple minor surgical operation on a man’s vas deferens which prevents sperms from entering the semen during ejaculation. A vasectomy is effective after about 3 months or after 20–30 ejaculations and a simple test (semen analysis) shows there are no more sperm in up-taker’s ejaculate [5].

Male sterilization unlike female sterilization (tubal ligation) can be done in the urologist’s office and a vasectomy procedure does not carry the risk of general anesthesia or intra-abdominal surgery. The procedure has a failure rate of about 0.05% [6, 7].

The procedure involves incision; occlusion or excision of a portion of the vas deferens, during the procedure, the vas deferens of a man are severed, and then tied or sealed [8]. The commonly used method in world is the no-scalpel. In Ghana, nearly all vasectomies are performed under local anesthesia using the no-scalpel technique [9].

In developing countries, vasectomy has long been considered a controversial method of contraception [10]. A study in Tanzania indicated that women lack trust in their partner’s fidelity after a vasectomy and therefore, wives play an important role in the decision of a vasectomy uptake by the husband [11]. In the United States of America, a study conducted by Bertotti in 2013 showed that some married and cohabiting women do not want to be sterilized themselves; they would prefer to avoid the health risks and emotional labor of sterilization but instead rely on their partners’ vasectomy. According to him although majority of the women approved of the use of male contraceptives by men, their attitude towards vasectomy by their spouses was however different. Nearly two-thirds (66.5%) of the women have disapproved the use of vasectomy by their spouses and this depends on the education level and the economic status of the women [12].

In the RESPONDS project in Uttar Pradesh, stories were shared about men who have undergone vasectomy recently [13]. It was a common decision for men to go for no - scalpel vasectomy (NSV) without discussing the matter with their wives and mothers, as they feared the women would try to dissuade them. The rate of acceptance is low in sub-Sahara Africa, where less than 0.1% of married women rely on a partner’s vasectomy [14]. In Ghana advocacy, has been done to try to improve vasectomy uptake; a report from The ACQUIRE project stated that one out of 1000 men in Ghana was using vasectomy as a family planning method [15]. Even though contraceptive prevalence is higher in urban communities, not much is known regarding the thoughts about vasectomy. In 2004, an awareness creation program on vasectomy service in Ghana, dubbed: “Get a Permanent Smile” was carried out in the Accra and Kumasi Metropolitan areas by The ACQUIRED project/EngenderHealth. The awareness campaign, used both macro and micro-media campaign strategies and it lasted for 4 months. During the intervention period vasectomy uptake increased from 18 to 81. For the period 2010–2013 (half year), the Accra Metropolitan Health Directorate recorded only 50 vasectomy procedures [16]. This statistic shows that vasectomy is not a popular family planning method in the Accra Metropolitan area.

This research sought to explore the perspectives of urban Ghanaian women on vasectomy.

### Methods

The study took place in Accra, the capital city of Ghana, largely an urban community but also has a few peri-urban communities. Accra is a cosmopolitan city that houses people from all the other regions of Ghana and beyond. The Accra metropolitan area has a total population of 1,848,614 from the 2010 population and housing census [17]. Accra has a variety of health facilities; a teaching hospital, four government hospitals, five quasi-government hospitals, seven polyclinics, two major Non – governmental Organisations (NGOs) clinics and a host of private health facilities that provide family planning services in the five health sub-metropolitan areas of Ablekuma, Ayawasu, Ashiedu -Keteke, Okaikoi and Osu Klottey.

The study was conducted from September to October 2013. Five focus group discussions were held with young adult women, aged 18–24 years and adult women aged, 25–49 years and each group had between 8 and 12 participants. The women were members of identified organized groups; youth groups, religious groups, social club groups, trader and corporate unions. The groups were selected by stratified purposive sampling. In total, forty-eight (48) participants partook in the study; twenty-three (23) young adult women and twenty-five (25) adult women (Additional file 1).

A discussion guide was used to elicit information from study participants. Two female field assistants were trained to assist with the data collection for the study. Two female field assistants were used for the data collection, due to the fact that the study was going to be held with females and female discussants would be more comfortable expressing their views on the subject with fellow women than with males. The discussions were held in Ga, Twi (two widely spoken Ghanaian languages in Accra) and English, depending on the language the participants were comfortable with, and were able to express themselves in. The discussion guides were originally written in English and were translated into Twi and Ga, and back into English by experts who were fluent in all these languages. During the sessions, the discussants were allowed to exhaust one topic before new topic was introduced and each session lasted 60–90 min. The sessions were held in private places that had been approved by the discussants. A key weakness of the study is that only female respondents were included.

The Committee on Human Research Publications and Ethnics, (CHRPE) of the Kwame Nkrumah University Sciences and Technology/Komfo Anokye Teaching Hospital approved the study. Leaders of the various groups provided administrative clearance for the study and assisted with the group compositions. Verbal consent was sought from each discussant and confidentiality was assured before each session.

An audio recorder was used to record the discussions and field notes were taken as well. Data were analyzed manually after transcribing and coding, and themes were sorted using thematic version 0.9.

### Findings

Majority of women opined that family planning decisions should be jointly taken by couples and not one partner’s decision - especially vasectomy. Although majority of the women may approve of the use of male contraceptives, their views on vasectomy by their spouses or partners were however different. Majority of women disapproved the use of vasectomy by their partners due to social and health reasons, however, some would approve of it if it was to their health benefit or if it would lead to economic gains in terms of a reduction in household size.

### Social perspectives

In Ghana, marriage and childbirth are considered an important aspect of one’s social standing. The discussants indicated that they would hesitate to ask their partners to adopt vasectomy mainly due to social factors.

In cases, such as divorce or separation or even death and in the event of remarriage, the new women will not have children of their own and they will be ridiculed in the society.

*“Assuming I am the husband and then my wife comes with this idea, I don’t think I will buy the idea because anything can happen, she might not even die but decide to leave me. If I see a young lady who has not given birth and I want to marry… She loves me and I also love her, marrying her without her own child will be like a hell for her so no matter what… I will not buy the idea. I will rather go for the natural way of spacing my children than going for this thing.” (Fire service personnel, 25–49 years)*

*“Men can easily change, because just in case there is divorce or separation and the man goes in for another woman who has not given birth before, how are they going to manage?”* (Policewoman, 25–49 years)*.*

Even though it is becoming a common practice to have fewer children, society still puts premium on the number of children one has.

*“Actually with this world which is full of uncertainty, many people may hesitate to go in for such an exercise because you are thinking that oh I am young, something can happen to me, my children and he remarries or something, he has to reproduce again.”*

(University student, 18–24 years)

Socially and religiously, partner cheating is frowned upon in Ghanaian communities, but even though this is not allowed, men are pardoned for cheating and society will normally blame the wives of such men for their conduct. Most of the respondents see vasectomy as a means for their male partners to cheat on women, while the women get blamed for the men’s unacceptable behaviors.

The behavior of vasectomized men has social connotations which affect the wives or their partners socially. The inability to make a woman pregnant after a vasectomy, “allows” vasectomized men to become promiscuous and this behavior affects their partners or their wives. Community members can even call the partners or wives of such men names, point accusing fingers at them, and blame the women for their partners’ or husbands’ cheating habits.

*“Yes, some men will frustrate you by having affairs with several other women because he knows he cannot make a woman pregnant.” (Policewoman, 25–49 years)*

*“This will be a license for the men to womanize indiscriminately; the woman (wife) will be disrespected because of her husband’s infidelity.”* (Hair dresser, 18–24 years)

Muslim wives cannot encourage their partners to opt for a vasectomy, due to the polygamous nature of the Islamic religion.

*“For my religion, he can marry another wife so if I should just end him or cut the sperms, if I am too old and he wants marry another woman how would the society even look at new woman…… they would think she is barren but me I don’t think I will go in for vasectomy”.* (University student, 18–24 years)

*“You know with we Muslims the men can marry up to four, so if you the woman gives birth to three children and tell the man that you are tired or you don’t wish to have any more children and he wants more he has to marry another woman; but that woman cannot give birth if the man has gone in for vasectomy and that will be very frustrating for the other wife because she is also a married woman who deserves to have her own children.” (*Muslim woman, 18–24 years)

### Health perspectives

Some study participants perceived that when men get vasectomized, they will be sleeping indiscriminately with other women without using protection and that can bring them (women) sexually transmitted diseases.

*“Some men knowing, they cannot impregnate women will take advantage of it and chase women around he might contract sexually transmitted diseases.”* (University Student, 18–24 years).

*“Men cannot be trusted in the sense that, he would assure you that you are the only woman in his life but when you delve into details you may find out that he is telling a lie, there are others. So if you are my husband and you do vasectomy without my knowledge we will sleep in separate rooms so we are not tempted, that you don’t infect me with any disease.”* (Market women, 25–49 years).

Other women were of the view that if their partners took up vasectomy, it would affect the women’s sexual health.

*What if he gets back from taken a vasectomy and he can’t perform anymore, my sexual health will be endangered oooo!* (Muslim Woman, 18–24 years)

*Me! (Hitting her chest) I wish they would not do it at all, because without it they are cheating already, so, what will happen if they do it (vasectomy)… I’m afraid of the disease aspects which can affect his performance.”* (Hair dresser, 18–24 years).

On the other hand, some women said, they could ask their men to take up vasectomy only when (the women) have a health condition or if it is on a doctor’s advice for their partners to have the procedure to the health benefit of the woman.

*“It is good when his wife has given birth a lot, going to the labor ward a lot of times comes with its own health problems in the future and so, he can go in for it to my benefit…. why not?”* (University Student, 18–24 years).

A few of the discussants held the view that if they were made to understand what the procedure entails and the uptake of vasectomy will not pose any health threat to their men’s health they will allow their partners to undergo the procedure.

*“Let’s say, I have like three children, and once the doctor assures me that it is problem- free procedure, I will allow him to do it. As a woman, I wouldn’t like to take up family planning because I learnt that it can make you bloat and could also make you grow lean, so that is the only reason why.” (Hair dresser, 18–24 years).*

Others also said that if vasectomy is recommended to their partners by a health professional or a medical doctor, then they will be comfortable with their partners’ uptake of vasectomy.

*“Because of technological advancement, I will encourage him to go for it on my behalf only if the doctor advices so.” (*Policewoman, 25–49 years*).*

### Economic reasons

This is a quote from one of the respondents*…. “Well! times are difficult now so if you have two or three children he has to do it in order not to give birth again.”* Throughout all the selected groups, there was consensus that vasectomy led to fewer children which reduces the financial burden on the family. Underneath the health and social benefits of vasectomy there was a strong economic motivation.

*“This family planning will help families to control expenses. Some women are highly fertile such that anytime they go near sex they become pregnant, for all you know the husband of such a woman might not be in a position to take good care of the family. So to such a family, it is good.” (Muslim woman, 18–24 years).*

*“Yes, times are hard ooo!! There is no money in…. some men will give birth with different women anywhere they find themselves, such that when they are dead and gone now these children will also become burdens on the woman, and I do not like that to happen to me.”* (Policewoman, 25–49 years)

*“As I am talking right now I have twins and another child from another woman that I am taking care of now because my husband is a womanizer. I am the one taking care of his three other children so when I tell him to go do vasectomy he will do it, because when he runs away the children will become my burden. I will call him and talk to him in the room at dawn, so that he will go and do it, things are hard for us now.”*

(Market Woman, 25–59 years)

The study also discerned that economic motivation is so strong an influencer such that, some respondents would tweak their religious or cultural beliefs in order to lessen the family’s financial burden of having many children.

*“The bible encourages us to give birth to as many children as we can but because of economic hardship these days, it is better to go in for the vasectomy”*

(Hair dresser, 18–24 years)

*“I may think that he wants to protect himself from having more babies because times are hard. Bible says if you have plenty children and you cannot look after them it is a sin.”* (Market woman, 25–49 years).

*“Sure, you see I spoke to my man, to look at the economy…let’s say we have two, if all of them are females, fine, if all of them are male, fine, if they are mixed, fine. Lucky for us, they are mixed. God has asked us to replenish the earth but the system is hard ooo.”*

(Fire service personnel, 25–49 years)

## Discussion

One of the benefits of vasectomy as a family planning method is the reduced financial burden on the family [18]. Throughout the focus group sessions, it was affirmed that the uptake of vasectomy leading to fewer children will reduce the financial burden on the family. Based on the general knowledge of the benefits of family planning by the women, they widely appreciated the fact that the up-take of vasectomy as a family planning method would help control the family expenditure. These are in cases where the husband is not in a position to take good care of the family. To such women, vasectomy it is good option. The study deduced that it is admissible that good health contributes directly to economic development in households and communities at large. The study also discerned that economic motivation is so strong an influencer such that, some respondents would ignore their religious or cultural beliefs in order to lessen the financial burden. A study conducted in Tanzania by Bunce and others, reported this from a respondent; “We decided ourselves although we are not allowed. It is the situation at home plus the benefits obtained by those who engaged us to join the services although my husband is a Roman Catholic catechist.” [19]. Despite these remarks, the study took in a high laxity in the use of vasectomy is attributable to more than one factor. Significant among these myriad of factors is the assertion that vasectomy makes men promiscuous. Family planning has been identified by the World Health Organization (WHO) as one of the six essential health interventions needed to achieve safe motherhood and by the United Nations Children’s Fund (UNICEF) as one of seven strategies for child survival [20]. In spite of all the health education and sensitization programs on vasectomy, it was understood in this study that quite an important number of women were very skeptical about the use of vasectomy on the grounds of likely health complications. Many studies indicate that vasectomy has no physical effect on a man’s virility, because it doesn’t change the testes’ production of the male hormone (testosterone), so the sex drive, potency, male characteristics, and sexual pleasure should be unchanged [21]. About 30% of men report improved sexuality after a vasectomy, most likely because the worry of pregnancy is eliminated [22]. Due to the inadequacy and general paucity of information available to individuals and communities about vasectomy, a large number of people remain highly skeptical when it comes to the point of decision. For instance, the women expressed sentiments such as vasectomy is a complicated method to them and they would not go in for such a complex method*.* The lack of clarity about the process and the general function of the male sexual organ leave the women concerned that their partners may endanger their partners’ health if they choose to use vasectomy. As vasectomy is seen as a reason for men to become promiscuous, women perceive its uptake will bring diseases (STIs or even HIV/AIDS) to them as the men sleep around indiscriminately without protection. The women were concerned about their health and so they would not allow their men to take up vasectomy. However, this study noticed that women would consider their partners undergoing vasectomy if it is recommended to them by a doctor.

### Conclusion

The findings from the study indicate that by and large, from the urban Ghanaian women’s perspective, they would allow their partners to take up vasectomy only because times are hard and also if it will benefit them (the women) health wise. They will disapprove of the uptake of vasectomy because the men will cheat on them and bring them diseases that will cost them (women) their health and also such behavior by their partners will bring society ridicule. In Ghana, vasectomy appears to be shrouded by well-crafted misconceptions, superstitions and illogical beliefs. Vasectomy advocates, family planning programmers and political/social/religious/traditional leaders could emphasize the economic gains that family planning (in general) brings to individuals, households and nations, while stressing the positives of vasectomy. Instead of using only vasectomized men as ‘brand’ ambassadors or satisfied clients to promote vasectomy, it would be advantageous to involve couples who use vasectomy as a family planning method.

## Additional file

Additional file 1: Composition of focus group discussion. (DOC 33 kb)

## Abbreviations

AIDS: Acquired Immune Deficiency Syndrome
CHRPE: Committee on Human Research Publications and Ethnics
HIV: Human Immunodeficiency Virus
ICPD: International Conference on Population and Development
NGOs: Non - governmental organizations
NSV: No–scalpel vasectomy
STIs: Sexually transmitted infections
UNICEF: United Nations Children’s Fund
WHO: World Health Organization
