# Supplementary material for: Perspectives of urban Ghanaian women on vasectomy
Source: Reprod Health. 2017 Feb 8;14:21. doi: 10.1186/s12978-017-0286-5 (PMC5299787; doi:10.1186/s12978-017-0286-5)
Supplement: Additional file 1: — Composition of focus group discussion. (DOC 33 kb) [file 12978_2017_286_MOESM1_ESM.doc]

**APPENDICES**

**Tables 1 Composition of Focus Group Discussion.**

| **HEALTH SUB- METROPOLITAN AREA** | **GROUP** | **AGE RANGE OF DISCUSSANTS** | **GROUP COMPOSITION** |
| --- | --- | --- | --- |
| ABLEKUMA | MUSLIM WOMEN | 18-24 | YOUNG ADULT WOMEN ONLY |
| ASHEIDU -KETEKE | FIRE PERSONEL | 25 – 49 | ADULT WOMEN ONLY |
| AYAWASU | UNIVERSITY STUDENTS | 18 – 24 | YOUNG ADULT WOMEN ONLY |
| OKAIKOI | MARKET WOMEN | 18 – 49 | MIXED AGED GROUP |
|  | HAIR DRESSER |  |  |
| OSU KLOTEY | POLICE WOMEN | 25 - 49 | ADULT WOMEN ONLY |

## Discussion Guide for Focus Group Discussions

**“*WHAT IF HE GETS A VASECTOMY AND HE CAN’T PERFORM ANYMORE”*: PERSPECTIVES OF URBAN GHANAIAN WOMEN ON VASECTOMY**

**.………………………………………………………………………………………………**

**SITE: MODERATOR:**

**NO. PARTICIPANTS: NOTE TAKER:**

**DATE: TRANSCRIBER:**

**START TIME: FINISH TIME:**

Introduction Questions

**Have you ever heard of any family planning method for men call vasectomy?**

**Supposing you do not want to have any more children, will you ask your partner to go for a vasectomy instead of you taking up any other family planning method?**

**Probing Questions.**

Will you recommend vasectomy to your husband? If yes, why? If no. why not?

What if your partner / spouse takes - up vasectomy without telling you?

What will be your reaction to his choice?

Why will you react in that way?
